# Cellular and Cytokine Correlates of Severe Dengue Infection

**DOI:** 10.1371/journal.pone.0050387

**Published:** 2012-11-29

**Authors:** Gathsaurie Neelika Malavige, Li-Chieh Huang, Maryam Salimi, Laksiri Gomes, S. D. Jayaratne, Graham S. Ogg

**Affiliations:** 1 Department of Microbiology, Faculty of Medical Sciences, University of Sri Jayawardanapura, Nugegoda, Sri Lanka; 2 Department of Medicine, Faculty of Medical Sciences, University of Sri Jayawardanapura, Nugegoda, Sri Lanka; 3 MRC Human Immunology Unit, Weatherall Institute of Molecular Medicine, Oxford NIHR Biomedical Research Centre and University of Oxford, Oxford, United Kingdom; 4 Department of Dermatology, Churchill Hospital, Oxford, United Kingdom; University of Rochester, United States of America

## Abstract

**Background:**

The occurrence of dengue haemorrhagic fever (DHF) is thought to result from a complex interplay between the virus, host genetics and host immune factors. Existing published data are not consistent, in part related to relatively small sample sizes. We set out to determine possible associations between dengue virus (DEN-V) NS3 specific T cells and cytokine and chemokine levels and the pathogenesis of severe disease in a large cohort of individuals with DHF.

**Methodology/Principal Findings:**

By using *ex vivo* IFNγ ELISpot assays we determined DENV-NS3 specific responses in patients with varying severity of DHF. Other cytokines produced by DENV-NS3 specific T cells were determined by using multiple bead array analysis (MBAA). We also determined the serum cytokine levels using MBAA, lymphocyte subsets and Annexin V expression of lymphocytes in patients with varying severity of DHF. Of the 112 DHF patients studied, 29 developed shock. Serum IL-10 and IP-10 levels positively and significantly correlated with T cell apoptosis while IL-10 levels inversely correlated with T cell numbers. In contrast, TGFß showed a very significant (P<0.0001) and positive correlation (Spearman’s R = 0.65) with the platelet counts, consistent with platelet release. We found that whilst patients with severe dengue had lower total T cell numbers, the DV-NS3 specific T cells persisted and produced high levels of IFNγ but not TNFα, IL-3, IL-13, IL-2, IL-10 or IL-17.

**Conclusions/Significance:**

Our data suggest that serum IL-10, TNFα and TGFβ differentially associate with dengue disease severity.

## Introduction

Dengue viral infections (DVI) have become one of the most important mosquito borne viral infections in the world and represent one of the major emerging infectious diseases. It is estimated that 2.1 million cases of dengue haemorrhagic fever (DHF)/dengue shock syndrome (DSS) occur every year resulting in 21,000 deaths [1]. Infection with any of the four dengue viruses may cause a wide spectrum of clinical features from asymptomatic disease, an undifferentiated febrile illness, dengue fever, DHF and DSS [2]. Infection can be from any of the four dengue serotypes which are closely related. Initial infection with a particular serotype is known as primary infection (PD), which is usually asymptomatic or results in mild disease manifestations, but severe disease can occur [3], [4]. However, subsequent infection with other serotypes (secondary dengue infections (SD)) carries an increased risk of more severe disease which can manifest in the form of DHF/DSS [4], [5].

Currently, the pathophysiology of dengue viral infections and factors that result in severe clinical disease are not well understood. The occurrence of DHF/DSS is thought to result from a complex interplay between the virus, host genetics and host immune factors [6]. Cross reactive memory T cells are thought to contribute to immunopathology by altering the cytokine profiles during SD and are also believed to be less effective in eliminating the newly infective virus serotype [7], [8]. Therefore, they are thought to lead to enhanced viral replication and thus severe clinical disease. Apoptosis of T cells have shown to occur in DHF, which could be a contributing factor for virus persistence and severe clinical disease [9], [10]. Indeed higher viral loads are thought to correlate with clinical disease severity [11]–[12]. Furthermore, it has been shown that individuals with severe clinical disease have prolonged viraemia than those with milder disease [13], [14]. Leucopenia has been shown to associate with DVI [15] and has been considered as a warning sign by the latest guidelines issued by the World Health Organization [16]. When compared to other febrile illnesses and influenza, neutrophil and lymphocyte counts have been shown to be significantly lower in DVIs [15]. Therefore, it appears that severe dengue is associated with prolonged viraemia, leucopaenia and an inappropriate virus specific immune response.

DHF is characterised by spontaneous bleeding and plasma leakage, which is the main contributing factor for poor disease outcome. Plasma leakage which usually lasts for around 48 hours (the critical period) is thought to be due to increased vascular permeability and endothelial dysfunction [6]. Massive immune activation of T cells [17]–[19], monocytes [20] and macrophages have been shown to produce unfavourable cytokines in large quantities which in turn cause endothelial dysfunction and increased vascular permeability. Many studies have investigated the cytokines patterns in serum samples in patients with DHF and these studies have shown that TNFα, IL-6, IL-10, IL-1β, IFN-γ, IL-4, IL-13, IL-7, GM-CSF, MIF, along with several other cytokines were elevated in patients with DHF when compared to dengue fever (DF) [21]–[24]. Interestingly, Chen *et al* have analysed various cytokine levels in serum samples in patients with DHF found that IL-6, IL-10 and MIF were elevated in those who died due to dengue infections, whereas there was no difference in levels of TNFα and IFNγ [21]. In contrast, a larger study carried out by Priyadarshani *et al* showed there was no difference in serum TNFα, in patients with DHF when compared to those with DF [25]. *In vitro* studies carried out on DV infected peripheral blood mononucleocytes (PBMCs), in a limited number of patients showed that IFNγ, TNFα, IL-2 and IL-6 were released early in the DV infected PBMC cultures, and later IL-4, IL-5 and IL-10 were produced. Based on these *in vitro* observations and by the cytokine patterns in serum samples of patients with DHF and DF, it was hypothesized that severe dengue could be associated with a Th1 to Th2 switch during dengue infection.

Despite these studies, the T cell profiles in patients with severe dengue are not clear. Furthermore, all these studies have compared the differences between DF and DHF, considering DHF as a single disease entity. The current dengue diagnosis, treatment, prevention and control guidelines by the World Health Organization, describes DHF as a disease with a wide clinical spectrum [16]. Although DHF is associated with plasma leakage and spontaneous bleeding, this does not invariably lead to DSS, which is characterised by severe plasma leakage leading to reduced pulse pressure (<20 mmHg) and reduced organ perfusion (severe dengue). Therefore, we set out to determine possible cytokines and cellular immune factors that may contribute to the development of severe dengue infection. We found that patients who developed DSS, had significantly lower T cell numbers when compared to those who did not develop shock and that serum IL-10 and IP-10 levels positively and significantly correlated with T cell apoptosis and with T cell numbers. Serum TGFß showed a significant (P<0.0001) and positive correlation (Spearman’s R = 0.65) with the platelet counts. We also found that the IL-10 and TNFα was not produced by NS3-specific T cells suggesting a different cytokine source. However, production of TGFß by NS3-specific T cells positively and significantly correlated with the production of IFNγ.

**Figure 1:**
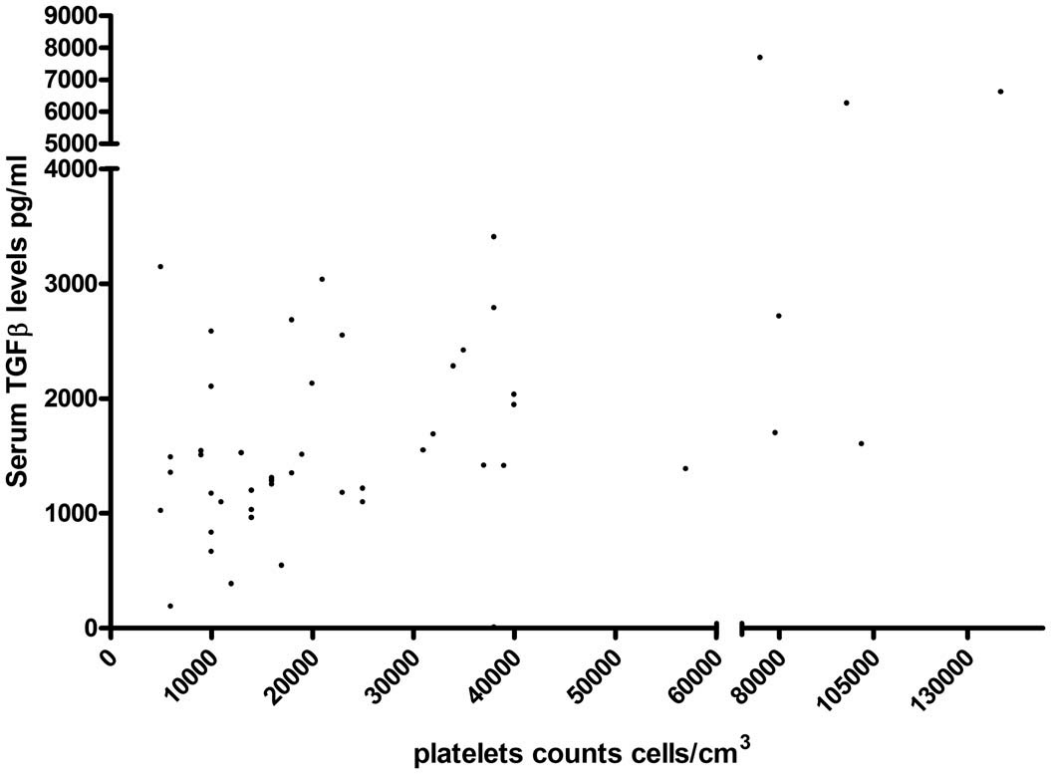
Correlation of serum TGFß (pg/ml) with the platelet counts in patients with DHF. The P<0.0001 and Spearman’s R = 0.65.

**Table 1:** Serum cytokine levels in patients with DHF who developed shock and those did not develop shock.

Cytokine levels	Patients with shockPg/mlN = 29Median (SD) and range	Patients without shockPg/mlN = 83	P value
TNFα	1.89 (9.14) 0 to 36.77	2.9 (169.4) 0 to 1168	0.35
IL-10	5.740 (15.14) 0.0800 to 71.00	5.17 (64.61) 0.0900 to 507.4	0.88
IL-17	0.34 (3.62) 0 to 19.52	0.51 (2.01) 0.0 to 13.33	0.83
IL-6	9.2 (54.94) 0.4700 to 276.5	9.7 (789.4) 0 to 6141	0.59
IP-10	3573 (7445) 453.0 to 29740	2263 (10414) 200.3 to 29740	0.26
MIP-1b	43.3 (62.75) 2.680 to 305.4	48.3 (134.9) 0.2400 to 795.7	0.89
TGF-ß	11015 (10866) 40.96 to 52860	12000 (11901) 1492 to 61390	0.81

**Table 2:** Correlation of serum TNFα levels with serum IL-10, IL-17, IL-6, MIP-1ß, IP-10 and TGFß levels.

Cytokine	Spearman’s R	P value
IL-10	0.2816	0.0026
IL-17	0.1948	0.0405
IL-6	0.4048	P<0.0001
MIP-1ß	0.4796	P<0.0001
TGFß	−0.07195	0.5881

## Methods

### Patients

112 patients with clinical features suggestive of dengue haemorrhagic fever and confirmed to be antibody positive (see below) were admitted to a general medical ward in a tertiary care hospital in Colombo were enrolled in the study following informed written consent. The study was approved by the Ethical Review Committee of the University of Sri Jayawardanapura and the Ethical review Committee of the University of Oxford. Serial recordings of their clinical features and laboratory investigations (platelet counts, haematocrits, white cell counts) were made until they were discharged from the hospital in order to determine the severity of dengue infection. Patients with mild dengue/dengue fever were excluded from the study, and only patients with DHF (those who had evidence of plasma leakage) were recruited. These patients were classified as having dengue with warning signs (moderately severe dengue) and severe dengue according to the 2009 WHO guidelines [16]. Patients with DHF with less than or equal to a pulse pressure of 20 mmHg were classified as having shock [16].

### Serology

DVI was confirmed by testing the serum samples which were collected after day 6 of illness with a commercial capture-IgM and IgG enzyme-linked immunosorbent assay (ELISA) (Panbio, Brisbane, Australia). The ELISA was performed and the results were interpreted according to the manufacturer’s instructions. This ELISA assay has been validated as both sensitive and specific for primary and secondary dengue virus infections [26]. Patients who only had dengue virus specific IgM were classified as having a PD infection while those who had a positive result for both IgM and IgG were classified as having a SD infection [16].

### Peptides

Synthetic 20 mer peptides overlapping by 10 amino acids which spanned the whole length of the DENV-3 NS3 protein were synthesized in house in an automated synthesizer using F-MOC chemistry. The purity of the peptides was determined to be greater than 90% by high-pressure liquid chromatography analysis and mass spectrometry. The synthetic NS3 20 mer peptides were pooled together to make a pool of NS3 peptides which represented the whole NS3 protein.

### *Ex vivo* ELISpot Assays

*Ex vivo* Elispot assays were performed as previously described [27] in 40 patients with acute dengue infection. For *ex vivo* ELISpot assays, 0.1×10^6^ PBMC were added to a final volume of 200 µl. Peptide was added at a final concentration of 10 µM. All peptides were tested in duplicate. PHA was always included as a positive control and an irrelevant peptide (SARS peptide) was included as a negative control. Background (cells plus media) was subtracted and data expressed as number of spot-forming units (SFU) per 10^6^ PBMCs. All peptides that induced an IFN-γ response of more than mean+3 standard deviations of the irrelevant peptide were considered positive.

**Table 3:** lymphocyte subpopulations in DHF patients who developed shock and those who did not develop shock.

Cell population	ShockN = 8Median (SD) and range Cells/mm^3^	No shockN = 27Median (SD) and rangeCells/mm^3^	P value
CD3+ T cells	269.8 (131.2)75.97 to 468.4	336.6 (428.5)141.5 to 2379	0.04
CD4+ T cells	182.9 (76.88)103.6 to 305.1	224.3 (262.1)116.5 to 1410	0.07
CD8+ T cells	120.4 (35.48)40.34 to 134.8	136.0 (166.5)20.42 to 903.8	0.07
B cells	36.0 (64.31)17.05 to 205.2	54.8 (46.44)10.15 to 202.6	0.16
Natural killer cells	47.25 (32.23)9.470 to 104.0	41.5 (58.33)1.610 to 233.2	0.42

### Multiplex Bead Array

Multiplex bead array assays were carried out as previously described [28]. Briefly, 25 µL of plasma samples (diluted 1∶4) and 50 µL samples of ELISpot supernatant (peptide stimulated and non-stimulated) incubated with anti-cytokine antibody-coupled beads (Bio-Rad) for 1 hour. For detecting TGF-β samples were incubated with 1N hydrochloric acid to be activated before 2-hour incubation with coupled beads. All incubations were performed at room temperature. After each step, complexes were washed 3 times in wash buffer (Bio-Rad) using a vacuum manifold. Beads were then incubated with biotinylated detector antibody for 1 hour, before incubation with Streptavidin-phycoerythrin for 30 min. Finally, complexes were resuspended in 125 µL of detection buffer, and 100 beads were counted during acquisition on the Luminex 200 (Bio-Rad). All experiments were performed in duplicates. The mean fluorescence intensity was analyzed and final concentrations were calculated in pg/mL.

**Table 4:** The correlation between serum cytokine levels and Annexin V expression on T cells and T cell numbers.

Cytokine	CD3+Annexin V expression	CD4+ Annexin V expression	CD8+ Annexin V expression	CD3+Numbers	CD4+Numbers	CD8+Numbers
IL-10	**0.3489** **0.0200**	**0.3696** **0.0157**	**0.2400** **0.0858**	**−0.3102** **0.0371**	**−0.2834** **0.0550**	**−0.3392** **0.0248**
TNFα	0.13580.2184	0.21840.4888	−0.0092570.4790	0.068330.3505	0.35050.3040	0.014730.4665
IL-17	−0.063780.3579	−0.014090.4680	0.057080.3723	−0.067420.3524	−0.094710.2971	−0.10520.2738
IP-10	**0.3828** **0.0116**	**0.3060** **0.0369**	0.13470.2202	−0.16900.1697	−0.20580.1215	−0.19600.1295
MIP-1ß	−0.17550.1566	0.15660.0753	**−0.3574** **0.0175**	0.12670.2377	0.095490.2956	0.11220.2605
IL-6	**0.3383** **0.0234**	0.20840.1148	0.10560.2730	0.10270.2817	0.28170.2693	0.11840.2492
TGFß	−0.29670.1625	−0.17580.2828	−0.15380.3079	0.46700.05	0.41760.0778	0.32420.1399

The top number on top of each column displays the Spearman’s R value and the number on the bottom row of each column displays the p value. Significant values have been made bold.

### Determining Absolute T Cell Numbers and Annexin V Expression

These experiments were carried out in 35 patients with DHF. Peripheral blood mononuclear cells (PBMC) were obtained from fresh heparinized blood by Ficoll-Hypaque density gradient centrifugation. To determine Annexin V expression on lymphocyte subsets PBMC were washed and stained with anti CD3 (FITC), anti CD4 (PerCP), anti CD8 (PE), anti CD20 (PerCP) and anti CD56 (PE) and then washed in annexin buffer (Biolegend) and stained using Annexin V (APC, Biolegend). Four-colour flow cytometric analyses were performed using FACSCalibur and Cell Quest software (Becton Dickinson, San Jose, CA).

### Determining Effect of IL-10 on Annexin V and Propidium Iodide (PI) Expression on T Cells

These experiments were carried out in 3 healthy dengue seropositive. PBMCs were incubated for 24 hours with human recombinant IL-10 (Peprotech, UK) was used at concentrations ranging from 10 pg/ml to 1000 pg/ml. After 24 hours incubation, the cells were washed and stained to determine Annexin V and PI expression on T cells. The PBMCs were initially stained with CD3 (APC) and CD8 (PE). The cells were then resuspended in annexin buffer (Biolegend, UK) and stained using Annexin V FITC (Biolegend) and PI (Sigma Aldrich [29]). Four-colour flow cytometric analyses were performed using FACSCalibur and Cell Quest software (Becton Dickinson, San Jose, CA).

### Statistical Analysis

Statistical analysis was carried out using PRISM version 4. As the data was not normally distributed, differences in means were compared using the Mann-Whitney t test (two tailed). To determine positive and negative associations, the Spearman’s correlation test was used (two tailed).

**Figure 2:**
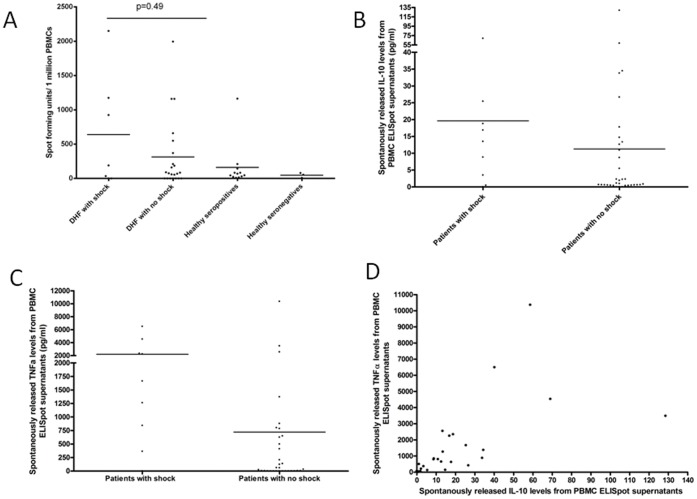
A: Ex vivo IFNγ ELISpot responses in patients with acute DHF who developed shock and those who did not develop shock and also in healthy dengue seropositive donors and dengue seronegative donors. B: Spontaneously released IL-10 levels (pg/ml) in PBMC ELISpot supernatants in patients with shock and those without shock The mean spontaneous release of IL-10 from PBMC in patients with shock was 19.65 (SD±21.28 pg/ml), and the mean levels in those who did not develop shock was 11.28 (SD±24.5) pg/ml (p = 0.08). C: Spontaneously released TNFα levels (pg/ml) in PBMC ELISpot supernatants in patients with shock and those without shock. The mean spontaneous release of TNFα from PBMC ELISpot supernatants in patients with shock was 2193(SD±2094) pg/ml and the mean levels ino those who did not develop shock was721.4S9D±1921) pg/ml). p = 0.009. D: Correlation of spontaneously released TNFα levels (pg/ml) and IL-10 levels (pg/ml) in the PBMC ELISpot supernatants in patients with DHF. Spearmans r = 0.88, p<0.0001.

## Results

### Clinical Characteristics and Severity of Dengue in the Study Population

Of the 112 patients with laboratory confirmed DHF, 77 (68.7%) were males. The mean age was 29.07 (SD±11.6, range 17 to 69 years). The mean duration of illness (day one was considered at the day they first developed fever) at the time of recruitment was 6.15 (SD±1.36) days. The patients were managed in the hospital for a median of 4 days. All patients had laboratory evidence of a rising haematocrit (evidence of plasma leakage) with a concurrent drop in platelet counts. 54 (48.2%) complained of abdominal pain and 44 (39.2%) had at least one bleeding manifestation. Based on the 2009 WHO diagnostic criteria, shock was defined as lowering of pulse pressure to 20 mmHg or less. Accordingly, 29 (25.5%) patients were classified as having shock. Abdominal pain, which is considered a warning sign by the latest WHO guidelines [16], was more frequent among patients with shock (62%) when compared to those who did not develop shock (43.4%) (Odds ratio 2.1, p = 0.09). There was no significant difference (p = 0.70) in the platelet counts of those who developed shock (mean 31.2, SD±30.8×10^9^/L) and those who did not develop shock (mean 33.5, ±32.9×10^9^/L). No difference (p = 0.14) was seen in the platelet counts between those who developed bleeding manifestations (mean 27.8, SD±27.9 ×10^9^/L) and those who did not develop any bleeding manifestations (mean 36.3, SD±34.7 ×10^9^/L). However, bleeding manifestations were commoner among patients with shock (62.1%), when compared to those who did not develop shock (31.3%) (Odds ratio 3.6, p = 0.004).

Based on the presence of dengue specific IgM but no IgG, 22 (19.6%) patients were found to have PD infection. The mean age of those with PD was 24.45 (SD±12.45) and those with SD was 29.9 (SD±12.45). 5 (22.7%) patients with PD and 24 (28.9%) of those with SD developed shock. Bleeding manifestations were present in 9 (40.9%) of those with PD and 35 (38.89%) of those with SD. However, only 3 (the 3 patients who developed shock) of those with PD had a significant bleeding manifestation (melaena, prolonged or large bleed).

### Cytokine Levels in Patients with DHF

We compared the serum levels of IL-10, IL-17, IL-6, TNFα, TGFß, MIP-1ß and IP-10 levels in patients with DHF who developed shock and those who did not. The median, standard deviation and range of these cytokine levels in these groups of patients is described in table 1. There was no statistically significant difference in the cytokine levels in patients with DHF who developed shock, when compared to those who did not (Table 1). However, a significant positive correlation was observed with TNFα levels and serum IP-10, IL-6, IL-17, IL-10 and MIP-1ß (table 2). No such association was seen between levels of TGFß and other cytokine and chemokine levels. Therefore patients who had high TNFα were also more likely to have higher levels of other cytokines, except TGFß suggesting that TGFβ may be differentially associated with DHF than the other cytokines. Moreover, TGFß showed a very significant (P<0.0001) and positive correlation (Spearman’s R = 0.65) with the platelet counts, consistent with platelet release (Fig. 1). However, none of the cytokines were associated with clinical disease severity.

### Lymphocyte Counts and Cytokine Levels

DHF is characterized by reduction of platelet counts, leucopenia and haemoconcentration due to fluid leakage. None of the tested cytokines showed any association with the total leucocyte counts or total lymphocyte counts. We also compared the levels of IL-10, IL-17, IL-6, TNFα, TGFß, MIP-1ß and IP-10 levels in patients with DHF with primary and secondary dengue infection. However, we did not find any significant difference in cytokine levels in patients with primary or secondary dengue.

95 (84.8%) of the patients had absolute lymphocyte counts <1500. There was no statistically significant difference (p = 0.45) in the lymphocyte counts, in those who developed shock (mean 663.8, SD±358.6) and those who did not develop shock (mean 860.2, SD±831.4). However, further analysis of lymphocyte subpopulations in 35 patients (7/35 had shock) showed that T cell numbers were significantly lower (p = 0.04) in those who developed shock (mean 279.2, SD±131.2 cells/mm^3^) when compared to those who did not develop shock (mean 472.6, SD±428.5 cells/mm^3^) (table 3). Although there was no statistically significant difference in CD8+ T cell numbers, CD8+ T cell percentages were significantly lower (p = 0.003) in those with shock (mean 14.19, SD±19.13) when compared to those who did not develop shock (mean 23.09, SD±8.18). However, no such difference was observed in B cell numbers and natural killer cell numbers in these 2 groups of patients (Table 3).

Serum IL-10 levels significantly and negatively correlated with absolute T cell numbers in these patients, suggesting that high IL-10 levels were associated with lower CD3+, CD4+ and CD8+ T cells. No such association was seen with B cell or NK cell numbers. Levels of other cytokines or chemokines were also not associated with T cell numbers or with B cell or NK cell numbers (table 4). Although not statistically significant (p = 0.05), TGFß levels appeared to positively correlate (Spearman’s R = 0.4670), with CD3+ T cell numbers, and CD4+ T cell numbers (p = 0.07, Spearman’s R = 0.4176).

**Figure 3:**
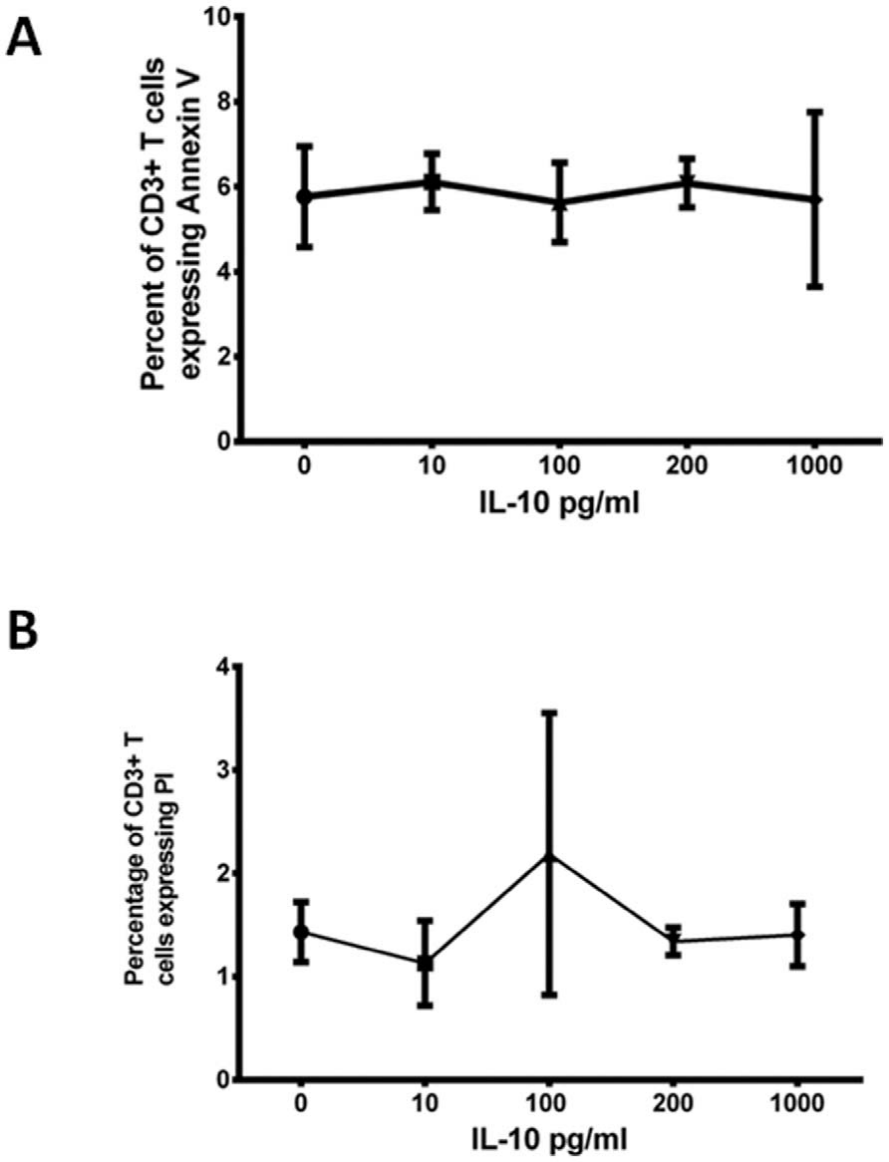
A: Percentage of CD3+ T cells expressing Annexing V following incubation of PBMCs with varying concentrations of IL-10 over a 24 hour period. The experiments were carried out in three dengue seropositive donors. B: Percentage of CD3+ T cells expressing Propidium iodide following incubation of PBMCs with varying concentrations of IL-10 over a 24 hour period. The experiments were carried out in three dengue seropositive donors.

### Cytokine Levels and Annexin V Expression by Lymphocytes

Serum IL-10 levels were found to significantly and positively correlated with Annexin V expression by CD3+ (Spearman’s R = 0.35, P = 0.02) and CD4+ T cells (Spearman’s R = 0.37, P = 0.01) but not significantly with CD8+ T cells (Spearman’s R = 24, P = 0.08). Apart from IL-10, IP-10 levels were also seen to be significantly and positively correlate with Annexin V expression in CD3+ and CD4+ T cells but not in CD8+ T cells, suggesting that high levels of IL-10 and IP-10 was associated with T cell apoptosis (table 4). Again although not significant, there was a trend towards TGFß levels negatively correlating with Annexin V expression on T cells.

### DENV-3 NS3 Specific Responses in Patients with Acute Dengue

*Ex vivo* IFNγ ELISpot assays were carried out in 40 of these patients with dengue infection. Of these 40 patients, 10 patients had developed shock, while the rest of the patients had DHF without any shock. There was a trend towards higher IFNγ DENV-3 NS3 specific responses in patients who developed shock (mean 639.3, SD±820.1) when compared to those who did not develop shock (mean 313, SD±513.1), but this was not statistically significant (p = 0.49) (Fig. 2A). The *ex vivo* IFNγ NS3 responses in patients with DHF were higher than in healthy dengue seropositive individuals, suggesting that NS3 specific responses are expanded in acute dengue infection (Fig. 2A) Although we did not observe any difference between TGFß production by NS3-specific T cell in those with shock and those who did not develop shock, TGFß levels positively and significantly correlated with IFNγ DENV-3 NS3 specific responses (Spearmans R = 0.50, p = 0.011).

We did not observe significant production of TNFα, IL-10, IL-4, IL-13, IL-17 by dengue NS3-specific T cells, suggesting a different source for these cytokines in the serum. Interestingly, PBMC incubated overnight in the absence of any further stimulation produced large amounts of TNFα, IL-10 and also TGFß (spontaneous cytokine release). Spontaneous release of TNFα from PBMC ELISpot supernatants was significantly higher (p = 0.009), in patients with shock (mean 2193, SD±2094 pg/ml) when compared to those who did not develop shock (mean 721.4, SD±1921 pg/ml) (Fig. 2B). Although not statistically significant (p = 0.08), spontaneous release of IL-10 from PBMC were higher in patients with shock (mean 19.65, SD±21.28 pg/ml), when compared to those who did not have shock (mean 11.28, SD±24.5 pg/ml) (Fig. 2C). However, no such observation was made with TGFß levels in the non-stimulated wells of PBMCs. The absence of TNFa and IL-10 production by the NS3-specific T cells, suggests that the TNFα and IL-10 may not be T cell derived.

Interestingly, spontaneous release of IL-10 from PBMCs showed a very significant and positive correlation with spontaneous release of TNFα from PBMCs (Spearmans r = 0.88, p<0.0001) (Fig. 2D). The spontaneous release of IL-10 (Spearmans r = 0.44, p = 0.008) and TNFα levels (Spearmans r = 0.48, p = 0.006) from PBMCs correlated positively and significantly with serum MIP-1ß levels. Again, spontaneous release of TGFß from from PBMCs did not correlate with either IL-10 or TNFα levels or serum MIP-1ß levels. Taken together our data suggests that the two immunosuppressive cytokines IL-10 and TGFß, possibly play different roles in the pathogenesis of dengue.

### Effect of IL-10 on T Cell Apoptosis

As IL-10 was found to be associated with T cell apoptosis in patients with acute dengue, we proceeded to find out if IL-10 causes T cell apoptosis. After incubating PBMCs of three healthy dengue seropositive donors with human recombinant IL-10 we did not find any difference in either Annexin V expression or PI expression in any of the individuals at varying concentrations of IL-10 (Fig. 3A and 3B).

## Discussion

We have investigated the possible associations between DEN-V NS3 specific T cells and cytokine and chemokine levels and the pathogenesis of severe disease in patients with acute dengue virus infection. We found that severe dengue was associated with a reduction of total T cells and an elevation of IL-10 and TNFα, which was not, produced by NS3-specific T cells. In addition, IL-10 was found to be associated with reduction of T cells and T cell apoptosis. IP-10 was also found to be associated with T cell apoptosis, but not directly with reduction of T cell numbers. Importantly, serum TGFß levels showed a very significant (P<0.0001) and positive correlation (Spearman’s R = 0.65) with the platelet counts consistent with platelet release (Fig. 1).

Many studies have shown that DENV-specific T cells in acute dengue infection are highly cross reactive and may be suboptimal in clearing the infecting virus serotype [8], [19], [30]. *In vitro* studies carried out by infecting PBMCs of two donors with the DV have shown that virus infected PBMCs initially produced IFNγ, TNFα and IL-2, and later, IL-4, IL-5 and IL-10 [31]. Another study which assessed the cytokines produced by T cells during ADI in 6 patients with DF and 3 patients with DHF showed that the IFNγ/IL-4 ratio was reduced in the 3 patients with DHF [32]. In addition, several studies had shown that Th2 type cytokines are elevated in the serum of patients with DHF [24], [33]. Based on this evidence it was earlier believed that DHF could be due to the development of an unfavorable Th2 type response instead of a more favorable Th1 type of response [34]. We found that although not statistically significant, *ex vivo* IFNγ DEN-3 NS3 responses, which are thought to be highly cross reactive [7], are elevated in patients with shock when compared to patients with DHF who did not have shock. T cell responses to NS3 protein have been found to be predominant and indeed it has been shown that the magnitude of NS3 IFNγ responses correlated well with disease severity [35]. Although we too found that high levels IFNγ was produced by DV-NS3 specific T cells, low levels of IL-10, TNFα, IL-4, IL-13 and IL-17 were produced by these T cells.

Interestingly, the IL-10 levels and TNFα production by PBMC after overnight incubation without any additional stimulation were significantly higher in patients with shock when compared to those patients with DHF who did not develop shock. Taken together, these results suggest that the high levels of serum IL-10 is being produced by other mononuclear cells such as monocytes. Human monocytes have been shown to be the main target of the DV during acute infection due to direct infection and also by antibody dependent infection enhancement [36]. Polymorphisms in the IL-10 promoter region have been shown to confer susceptibility to severe dengue infection by production high levels of IL-10 in DV infected monocytes [37]. Previous studies have shown that patients with DHF had lower T cell numbers [38]. Others have found that serum IL-10 levels are higher in patients with DHF and particularly in those who succumbed to their illness [21], [22], [39]. We also found that T cells numbers negatively correlated with serum IL-10 levels, while apoptosis of T cells positively correlated with both serum IL-10 and IP-10 levels. IL-10 levels have also been shown to be associated with severe disease in patients with other virus infections such as influenza and related to neurological complications [40]. Collectively, these data suggest a possible role of IL-10 in the pathogenesis of severe dengue infections.

A higher rate of apoptosis of PBMCs has been described in patients with DHF when compared to those with DF [9], [10]. Studies carried out in a limited number of patients have shown that there is massive apoptosis of T cells in patients with DHF [10]. We too found that T cells preferentially underwent apoptosis in patients with DHF, which was found to be associated with serum IL-10 levels. Serum IL-10 levels also negatively correlated with T cell numbers. IL-10 has shown induce apoptosis of highly activated T cell by Fas-FasL pathyway in patients with systemic lupus erythematosis (SLE) [41]. However, IL-10 does not appear to induce T cell apoptosis in the levels found in patients with acute dengue infection (10–1000 pg/ml). Therefore, although IL-10 is associated with T cell apoptosis, it appears that both are markers of severe dengue possibly independently associated with each other. Viral loads have shown to be higher in patients with DHF when compared to those with DF [12] and also it has been shown that individuals with severe clinical disease have prolonged viraemia than those with milder disease [13], [14], [42]. Therefore, it appears that low T cell numbers due to T cell apoptosis could be associated with an impaired DV-specific T cell response leading to delay in clearing of the virus.

A previous study has showed TGFß1 levels were highest in patients with severe DHF and were lowest in patients with dengue fever [43]. However, we did not find any significant difference in serum TGFß levels in those with shock (mean 15194, SD±11901 pg/ml) than those who did not develop shock (mean 15131, SD±10866). We found that TGFß showed a very significant (P<0.0001) and positive correlation (Spearman’s R = 0.65) with the platelet counts, consistent with platelet release. We also did not observe any difference between TGFß production by NS3-specific T cell in those with shock and those who did not develop shock. However, TGFß levels in peptide stimulated ELISpot supernatants positively and significantly correlated with IFNγ DENV-3 NS3 specific responses. Therefore, it is possible that the immunosuppressive cytokines, IL-10 and TGFß play different roles in the pathogenesis of dengue infections.

In summary, we found that patients with severe dengue had lower T cell numbers but that DV-NS3 specific T cells produced high levels of IFNγ but not IL-3, IL-13, IL-2, IL-10 or IL-17. In addition, T cells were also seen to undergo apoptosis, which positively correlated with serum IL-10 and IP-10 levels. Although we did not observe a significant association with serum TGFß levels and T cell numbers or apoptosis, serum TGFß levels positively correlated with platelet counts. Therefore, it would be now be important to determine the possibly contradictory roles of IL-10, IP-10 and TGFß in the pathogenesis of severe dengue infections.
